# Therapeutic targets for rheumatoid arthritis: lessons from animal models

**DOI:** 10.1186/ar3573

**Published:** 2012-02-09

**Authors:** Yoichiro Iwakura, Shinobu Saijo, Susumu Nakae, Noriyuki Fujikado, Harumichi Ishigame, Masanori Murayama

**Affiliations:** 1 Center for Experimental Medicine and Systems Biology, Institute of Medical Science, University of Tokyo, Minato-ku, Tokyo 108-8639, Japan; 2 Core Research for Evolutional Science and Technology (CREST), Japan Science and Technology Agency, Saitama 332-0012, Japan

## 

We have generated two RA models, human T-cell leukemia virus type I (HTLV-I) transgenic mice and IL-1 receptor antagonist (Ra)-deficient (KO) mice, to elucidate the pathogenic mechanisms of the disease. Both models spontaneously developed arthritis closely resembling that of RA in humans. We found that TNF-, but not IL-6-, deficiency suppressed development of arthritis in IL-1Ra KO mice, while IL-6 but not TNF was involved in the HTLV-I transgenic mouse model [1]. IL-17 was important in both models. These observations suggest that pathogenic roles of IL-6 and TNF are different and both TNF, IL-6, and IL-17 are good targets for therapeutics.

We found that the expression of C-type lectin receptor (CLR) genes was augmented in the affected joints of these models using DNA microarrays. Dendritic cell immunoreceptor (DCIR) is one of such CLRs with a carbohydrate recognition domain in their extracellular carboxy terminus and an ITIM in its intracellular amino terminus. Because human shared syntenic locus containing the *Dcir *gene is linked to several autoimmune diseases including RA and SLE, we have generated *Dcir *KO mice to examine the roles of this gene in the immune system. We found that aged *Dcir *KO mice spontaneously developed sialadenitis and enthesitis associated with elevated serum autoantibodies [2]. DCs were excessively expanded in *Dcir *KO mice after aging. *Dcir *KO mouse-derived bone marrow cells (BMCs) differentiated into DCs more efficiently than did wild-type BMCs upon treatment with GM-CSF, owing to enhanced STAT-5 phosphorylation. These findings indicate that DCIR is crucial for maintaining the homeostasis of the immune system, suggesting that Dcir is one of novel targets for the treatment of RA.

We have also found that the expression of *Muratin1*, which encodes uncharacterized and secreted protein, is specifically up-regulated in affected joins of both models. Interestingly, the development of collagen-induced arthritis was markedly exacerbated in *Muratin1 *KO mice. I would like to discuss the roles of Muratin-1 in the development of arthritis.
